# *SIRT2*-dependent *CPT1A* deacetylation in macrophages inhibits periodontitis

**DOI:** 10.3389/fphar.2025.1574141

**Published:** 2025-06-18

**Authors:** Yixuan Jiang, Xiu Yao, Zhizhong Jiang, Xiaomeng Liu, Boyuan Sun, Zhengyu Guan, Lin Zhou, Hongjiao Li

**Affiliations:** ^1^ Department of Stomatology, Xinhua Hospital Affiliated to Shanghai Jiao Tong University School of Medicine, Shanghai, China; ^2^ Department of Oral Implantology, Shanghai Stomatological Hospital, Fudan University, Shanghai, China; ^3^ Department of Pediatrics, The First Affiliated Hospital of Naval Medical University, Shanghai, China

**Keywords:** *CPT1A*, *SIRT2*, periodontitis, macrophage, deacetylation

## Abstract

**Introduction:**

Periodontitis is intricately related to systemic disorders and exerts a negative impact on quality of life. Recent studies have suggested a potential association between periodontitis and fatty acid oxidation (FAO), a key metabolic process involved in energy production and cellular function. However, the molecular mechanisms underlying this relationship remain insufficiently understood. This study aims to explore the role of carnitine palmitoyltransferase 1A (*CPT1A*), a pivotal enzyme in fatty acid oxidation (FAO), in the pathogenesis of periodontitis.

**Methods:**

The involvement of FAO in periodontitis was validated through bioinformatics analysis and quantitative real-time polymerase chain reaction (qRT-PCR). The anti-inflammatory effects of the *CPT1A* inhibitor Etomoxir (ETO) were assessed by qRT-PCR and Western blot analysis. The interaction between Sirtuin-2 (*SIRT2*) and *CPT1A* was confirmed via Chromatin Immunoprecipitation (ChIP)-qPCR. An experimental model of periodontitis was induced using silk ligation, and the effects of *CPT1A* inhibition on periodontitis were evaluated in mice treated with ETO. Micro-Computed Tomography (micro-CT) and histological analyses were employed to assess the impact of *CPT1A* inhibition on tissue architecture and inflammatory response in the periodontal tissues.

**Results:**

ETO reduced the expression levels of TNF-α, IL-6, IL-1β, NF-κB, and MAPK. Furthermore, it decreased cementoenamel junction-alveolar bone crest (CEJ-ABC) distance, immune cell infiltration in gingival tissues, and the expression levels of iNOS and p65. Additionally, ChIP-qPCR further confirmed the interaction between Sirtuin-2 (*SIRT2*)-*CPT1A*, thereby impacting the acetylation levels of *CPT1A* and decreasing *CPT1A* activity.

**Conclusion:**

Overall, these findings demonstrate that *SIRT2* binds to and deacetylates *CPT1A*, thereby inhibiting osteoclast differentiation and concurrently alleviating inflammation in periodontal tissues during experimental periodontitis progression.

## 1 Introduction

Periodontitis is a prevalent condition characterized by the irreversible immuno-inflammatory deterioration of tissues that support tooth structure and remains the leading cause of tooth loss in adults (Cai et al., 2024; Tobita and Mizuno, 2010; Slots, 2017). Globally, over 1 billion individuals suffer from severe periodontitis (Nascimento et al., 2024). In China, fewer than 10% of adults are periodontally healthy, with over 50% presenting with periodontal pockets and over 90% of individuals suffering from periodontal disease (Kwon et al., 2021; Dannewitz et al., 2021). As is well documented, periodontitis is intricately related to systemic disorders such as metabolic syndrome, therefore compromising human health and imposing a significant burden on global healthcare systems (Czesnikiewicz-Guzik et al., 2019; Jiao et al., 2021; Thongyim et al., 2024). Our current understanding of the pathogenesis underlying periodontitis remains limited, and studies have primarily focused on oxidative stress, flora imbalance, metabolic disorders, etc. At present, there are no effective pharmacological agents approved for the prevention or treatment of this condition. Thus, there is a pressing need to conduct systemic and integrative studies to identify effective and safe drugs for the treatment of periodontitis.

Periodontitis is hallmarked by immune cell infiltration and osteoclast differentiation. Macrophages play a decisive role in innate immunity in the initial host defense against microorganisms, especially in periodontitis (Zhang et al., 2024; Li et al., 2023). Additionally, recent studies based on rodent models have established that macrophage-mediated inflammation is closely associated with a reduction in Mitogen-Activated Protein Kinase (MAPK) activity (Ye et al., 2022; Shen et al., 2024). Activation of the MAPK signaling pathway is also closely associated with FAO (Yang et al., 2021). Thus, elucidating the metabolic transformation of macrophages in periodontitis may offer novel insights into the pathogenesis of periodontitis. FAO, the process by which free fatty acids (FFAs) are converted into fatty acyl-CoA esters, plays an essential role in mammalian FA metabolism. A marked increase in FAO appears to drive the shift in energy metabolism in periodontitis, promoting the production of reactive oxygen species, prostaglandins, and leukotrienes, all of which contribute to the ongoing inflammatory response in periodontal tissues (Liu et al., 2023). Notably, *CPT1A* is the rate-limiting and targeting enzyme in FAO. Numerous studies have established its relevance in inflammatory diseases (Chung et al., 2024; Park et al., 2021; Xu et al., 2024). For example, *CPT1A* promotes oxidative stress and inflammation in liver injury via modulating the Nrf 2/HO-1 axis (Luo et al., 2021). Moreover, its downregulation exerts protective effects against chronic ulcerative colitis by inhibiting peroxisome proliferator-activated receptor α (PPARα) expression (Chen et al., 2022). The introduction of constitutively active *CPT1A* in cultured macrophages inhibits the establishment of a palmitate-triggered inflammatory phenotype (Miguel et al., 2021). At the same time, macrophage phagocytosis and inflammatory phenotype are contingent upon intracellular lipid accumulation and *CPT1A* expression (Liu et al., 2024). Thus, we hypothesize that *CPT1A* may play a key regulatory function in chronic inflammatory diseases such as periodontitis by regulating macrophage FAO.

Previous studies have concluded that periodontitis is closely related to protein post-translational modifications (PTMs) such as acetylation and methylation (Jurdzińsk et al., 2020). In experimental periodontitis, a higher acetylation level of H3K9 was observed in gingival epithelial tissues. Nonetheless, the epigenetic mechanism underlying *CPT1A* histone modification in the development of periodontitis remains elusive despite the critical role of histone PTMs in the regulation of *CPT1A* (Montenegro-Navarro et al., 2023).

Members of the Sirtuin protein family contain NAD^+^-binding domains and have garnered extensive attention for their role in alleviating metabolic diseases such as diabetes mellitus (Wu et al., 2022; Mesa et al., 2019; Liu et al., 2021; Alamoudi et al., 2024). *SIRT2*, first identified in yeast, is a member of the Sirtuin family of nicotinamide adenine dinucleotide (NAD+)-dependent deacetylases with histone deacetylase activity. In addition, it participates in various cellular processes, including metabolism and bacterial infections (Rothgiesser et al., 2010; Jayasena et al., 2016). Li et al. (2023) discovered that it could protect against hepatic steatosis. Furthermore, *SIRT2*-deficient mice were more susceptible to obesity caused by a high-fat, high-cholesterol, and high-sucrose diet, which exacerbated steatohepatitis. In addition, *SIRT2* has been demonstrated to inhibit the activity of NLRP3 inflammatory vesicles and modulate the acetylation of p65, thereby playing a regulatory function in inflammatory processes (Liu et al., 2021; Yan and Horng, 2020). However, its role in FAO, especially *CPT1A*, remains underexplored.

Thus, this study aimed to investigate the role of *CPT1A* in experimental periodontitis through the use of both animal models and *in vitro* experiments to offer novel insights and potential strategies for the treatment of the condition.

## 2 Materials and methods

### 2.1 Bioinformatics analysis

The GSE27993 dataset on periodontitis, sourced from the Gene Expression Omnibus database (GEO database) of NCBI, was utilized to identify DEGs. It contains gene expression profiles from the periodontal ligaments of five patients with healthy periodontal ligaments and five patients with periodontitis. DESeq software was employed for differential gene expression analysis, with the screening criteria set at Padjust ≤0.05 and |logFC| > 1. GO functional and KEGG pathway enrichment analyses were performed on the differentially expressed genes using the R software. GO functions comprised biological processes (BP), cellular components (CC), and molecular functions (MF). The hypergeometric test was employed to conduct enrichment analysis. Significant enrichment was defined as an FDR-corrected P value of 0.05 or lower. Gene ontology (GO) and Kyoto Encyclopedia of Genes and Genomes (KEGG) analyses were conducted to investigate the potential functions of proteins and signaling pathways.

### 2.2 Reagents

Lipopolysaccharide derived from Porphyromonas gingivalis (P.gingivalis-LPS), a major pathogenic factor for periodontitis (cat# SMB00610), was purchased from Sigma-Aldrich (CA, United States). *CPT1A* inhibitor ETOMOXIR (cat# S41677) was procured from Yuan Ye (Shanghai, China). The *SIRT2* inhibitor AKG2 (cat# S7577) was acquired from Selleck (TX, United States).

### 2.3 Cell treatment

The mouse leukemic monocyte/macrophage cell line RAW 264.7 was acquired from the Typical Culture Collection Center of the Chinese Academy of Sciences (CAS). Bone marrow-derived macrophages (BMMs) were prepared as described previously (Yu et al., 2025), and murine M-CSF (25 ng/mL, R&D, United States) was added. All cell lines were cultured in an incubator (37°C, 5% CO2) (Thermo, Massachusetts, United States) with high-glucose DMEM (BasalMedia, Shanghai, China) supplemented with 10% FBS (SuperCulture, Shenzhen, China), 100 Units/mL penicillin, and 100 μg/mL streptomycin (Beyotime, Shanghai, China). *Porphyromonas gingivalis* (*P.gingivalis*) is the main causative agent of periodontitis, whilst its derived LPS is a key pathogenic factor for periodontitis (Dong et al., 2024; Zhu et al., 2024). To simulate inflammation *in vivo*, 1 μg/mL P. gingivalis-LPS was added to the culture medium of the model group for 3 h, as described in a previous study (Xu et al., 2020). To investigate the effects of ETO on LPS-induced inflammation, RAW264.7 cells were exposed to 1 μg/mL ETO or DMSO (vehicle control) for 30 min or 60 min.

### 2.4 Cell viability assay

RAW264.7 cells were treated with varying concentrations of ETO for 24 h. Next, the medium was replaced with 100 µL of 10% CCK-8 solution (Beyotime, Shanghai, China), and cells were incubated at 37°C in the dark. Absorbance was measured at 450 nm using a microplate reader (Bole Life Medical Products, Shanghai, China) to assess cell viability.

### 2.5 Quantitative reverse-transcription polymerase chain reaction (qRT-PCR)

Total RNA was extracted using an RNA extraction kit (Feijie Biology, Shanghai, China), and cDNA was prepared from 1,000 ng total RNA using the PrimeScript RT Reagent Kit (R333, Vazyme, Nanjing, China). Subsequently, qRTPCR was performed using the SYBR-Green PCR Master Mix Kit (RR420A, Takara, Japan). For data normalization, the housekeeping gene β-actin was used as the internal control. Relative gene expression levels were calculated using the 2^−ΔΔCT^ method. Primers specific to the tested genes were designed using PrimerBank (https://pga.mgh.harvard.edu/primerbank/). The primer sequences used in this study are as follows:

**Table:** 

Target	Primers	Sequences (5′–3′)
β-actin	Forward	ACA​TCC​GTA​AAG​ACC​TCT​ATG​CC
	Reverse	TAC​TCC​TGC​TTG​CTG​ATC​CAC
TNF-α	Forward	GGC​GTG​TTC​ATC​CGT​TCT​C
	Reverse	CTT​CAG​CGT​CTC​GTG​TGT​TTC​T
IL-1β	Forward	TTC​AGG​CAG​CAG​TAT​CAC​TC
	Reverse	GAA​GGT​CCA​CGG​GAA​GAC​AC
IL-6	Forward	GGAGCCCACAGAACG ATAGTCAA
	Reverse	TGT​CAC​CAG​CAT​CAG​TCC​CAA​GAG​G
APOD	Forward	TCA​CCA​CAG​CCA​AAG​GAC​AAA
	Reverse	CGT​TCT​CCA​TCA​GCG​AGT​AGT
MMP8	Forward	TGC​CAC​GAT​GGT​TGC​AGA​G
	Reverse	AGG​CAT​TTC​CAT​AAT​CCC​CAT​TG
LTF	Forward	TGA​TGC​CAT​GAC​TCT​TGA​TGG​T
	Reverse	TCT​TTG​GTC​CCG​TAG​ACT​TCA​G
IRF4	Forward	TCC​GAC​AGT​GGT​TGA​TCG​AC
	Reverse	CCT​CAC​GAT​TGT​AGT​CCT​GCT​T

### 2.6 Western blot analysis

RAW264.7 was lysed in RIPA buffer (Beyotime, Shanghai, China) containing phosphatase and protease inhibitors (Beyotime, Shanghai, China). Protein concentration was quantified using a BCA Protein Assay Kit (A55860, Thermo Fisher Scientific, Massachusetts, United States). The extracted proteins were separated by 8%–12% SDS-PAGE, transferred to polyvinylidene fluoride (PVDF) membranes (Millipore, Darmstadt, Germany), and subsequently blocked with 5% BSA for 2 h. Next, the membranes were incubated with the primary antibodies against JNK, p-JNK, p-ERK, ERK, p-p38, p38, p-p65, NF-κB p65 (1:1,000, Cell Signaling Technology, United States), and β-actin (1:10,000, ProteinTech, United States) overnight at 4°C in a refrigerator and then incubated with the secondary antibody (1:8,000, ProteinTech, United States) diluted in 1 × TBST solution at room temperature for 1 h. Protein bands were detected using a chemiluminescence system (Tanon-Bio, 4,600) and quantified using ImageJ 8.0 (Imagesoftware, Bethesda, MD, United States).

### 2.7 Specimen collection and preparation

Gingival tissue biopsies from five healthy individuals and five periodontitis patients were collected from surgical procedures performed at our hospital between 1 January 2023, and 1 June 2024. The specimens were primarily obtained during gingivectomy procedures conducted as part of tooth extractions. All participants provided informed consent in accordance with the new classification established at the 2018 periodontal workshop. Immediately following surgical resection, the fresh tissue samples were fixed in 4% paraformaldehyde solution for 24 h.

### 2.8 Chromatin immunoprecipitation (ChIP)-quantitative PCR

The cells were seeded in 10 cm plates, divided into the LPS and AGK2+LPS groups, and incubated for 24 h. The LPS group was treated with DMEM solution containing LPS (1 μg/mL), whereas the AGK2+LPS group was treated with DMEM containing AGK2 (1 µM), following which LPS was added to achieve a final concentration of 1 μg/mL. Afterward, a ChIP Kit (P2080S, Beyotime, China) was employed for the ChIP assays. Formaldehyde was used to cross-link cells at a final concentration of 1%. Thereafter, 1x glycine solution was added to terminate the cross-linking reaction. Then, DNA was sonicated to shear DNA to a size of approximately 200–1,000 bp. Afterward, the collected samples were purified using a DNA kit (Beyotime, Shanghai, China). Chromatin extracts containing sheared DNA fragments were IPed with specific antibodies (or nonspecific IgG) overnight at 4°C. ChIP-grade Protein A/G Magnetic beads were added, and the resulting mixture was incubated for 4 h at 4°C. Antibodies used for immunoprecipitation included H3K27ac and H3K9ac, with IgG serving as a negative control (Jingjie Bio, China). Thereafter, the acquired DNA was purified and subjected to qPCR (Beyotime, Shanghai, China). Data were analyzed using the CT method, and the results were expressed as % input DNA. ChIP-qPCR values were calculated using the following formula: % input recovery = [100/(input fold dilution/bound fold dilution)] × 2 (input CT-bound CT). The DNA fragments were analyzed by qRT-PCR using the following primer pairs:

**Table:** 

Target	Primers	Sequences (5′–3′)
*CPT1A* Promoter1	Forward	GGT​CTC​ATA​GCC​AAG​TCC​CC
	Reverse	CTA​GCC​TCC​TTT​CCG​GTT​GG
*CPT1A* Promoter2	Forward	CAT​GCA​TGC​AAC​CCT​ACG​AT
	Reverse	TCA​AAT​TGT​CCT​TGG​CCA​CAT​A
*CPT1A* Promoter3	Forward	GCA​TCA​TTA​TAC​CCT​CTT​TAT​GTG​G
	Reverse	ACA​AAC​CAC​AAC​TGG​GGA​TG
*CPT1A* Promoter4	Forward	CGG​GAA​CCA​AAC​TGT​GGT​CTT
	Reverse	GAG​TTG​GAG​GAA​ATT​GCT​CAG​TAA​C
*CPT1A* Promoter5	Forward	ACG​TAT​CCA​TCG​TGA​GCA​GC
	Reverse	GAA​CTG​GCA​CCA​AGG​CTA​GA
*CPT1A* Promoter6	Forward	TTC​TGA​TGT​TGT​CTC​CGC​CC
	Reverse	TTT​GCG​TCC​CTC​TGG​ATT​GG
*CPT1A* Promoter7	Forward	ACC​GGT​TAG​GAC​TGG​GGT​TC
	Reverse	GCT​CTA​GTG​TCA​CCT​CTT​GCT
*CPT1A* Promoter8	Forward	AGT​TAC​ACC​CAA​CAA​TCG​CCT
	Reverse	AAG​TTC​CCC​TTG​ATC​CCG​C

### 2.9 Animal

Male C57BL/6 mice aged 8 weeks (Charles River, Beijing, China) were used in this study. The mice were housed under specific pathogen-free conditions, with a temperature range of 22°C–26°C and humidity of approximately 40%–60%. They were provided *ad libitum* access to food and water, and no drugs other than ETO were administered during the experiment. All animal experiments were conducted following the protocol approved by the Animal Ethics Committee of Xinhua Hospital, Shanghai Jiao Tong University School of Medicine (XHEC-NSFC-2019-240). The animal procedures were performed in accordance with the ARRIVE guidelines (Animal Research: Reporting of *In Vivo* Experiments).

### 2.10 Animal model preparation and experimental grouping

**FIGURE 1:**
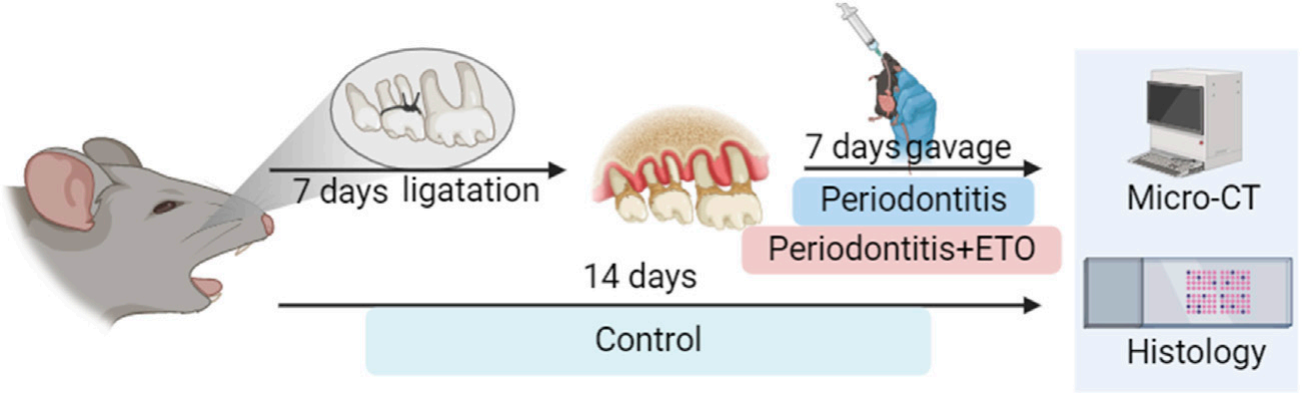
Experimental model of ETO treatment and experimental periodontal inflammation in mice.

A total of 18 mice were acclimatized to the laboratory environment 7 days before the experiment. Subsequently, they were randomLy assigned to three groups: (1) control, (2) model (3) ETO (n = 6 per group) using the random number table method. The control group comprised normal untreated mice, while the model group was subjected to periodontitis and administered vehicle control (corn oil). The ETO group was subjected to periodontitis and treated with ETO. For experimental periodontitis induction, the mice were anesthetized via intraperitoneal injection of ketamine and xylazine. The experimental periodontitis model was established using the ligation method, which has been widely used in previous studies (Hiyari et al., 2018; Huang et al., 2022; Yu et al., 2016; Kang et al., 2024). Briefly, a 4–0 silk thread was ligated around the second M on the left side of the mouse’s maxilla. The right side of the maxilla served as a control. After 7 consecutive days of observation, the mice were gavaged with ETO (80 mg/kg/d) (Cabrero et al., 2003) (Figure 1). On the 14th day, the mice were euthanized via assisted cervical dislocation after CO_2_ inhalation, and the maxillae of each mouse were collected and hemisected. The included maxillae were fixed with 4% paraformaldehyde for alveolar bone loss assessment and histological analysis, and the right maxillae were used as a control.

### 2.11 Micro-Computed Tomography (Micro-CT) analysis

To assess alveolar bone loss, the maxillae, including periodontal tissues and bone, were scanned using a Micro-CT (µCT50, Scanco, Zurich, Switzerland). The X-ray tube voltage of the system was set to 70 kV, with a current of 200 mA and an exposure time of 300 ms. Parameters such as bone mineral density (BMD) and bone volume/total volume (BV/TV) were determined. The distance between the cement-enamel junction (CEJ) and the alveolar bone crest (ABC) was measured and averaged using ImageJ 8.0 (Image software, Bethesda, MD, United States).

### 2.12 Histological analysis

The maxillae were decalcified in 10% ethylenediaminetetraacetic acid (EDTA). Then, they were dehydrated in ethanol, embedded in paraffin, and sectioned into 5-μm-thick sections. Lastly, morphological changes were examined and evaluated independently by two blinded researchers using an Olympus microscope (Nikon, Tokyo, Japan).

### 2.13 Hematoxylin and eosin

Sections were deparaffinized and hydrated. Following this, H&E staining was performed to evaluate the degree of inflammatory infiltration and histopathological changes in periodontal tissues using the H&E staining kit (G1120, Solarbio, Beijing, China). Images were captured under a light microscope (Olympus, Tokyo, Japan).

### 2.14 Immunohistochemical staining

Sections were deparaffinized and hydrated, followed by standard antigen retrieval and blocking procedures. The mice sections were then incubated with primary antibodies specific to *CPT1A* (Abconol, China, 1:200), *SIRT2*, iNOS, and p65 (Abcam, United States, 1:200) at 4°C overnight. The patients sections were then incubated with primary antibodies specific to *SIRT2* (Abcam, United States, 1:200). The samples were then incubated with secondary horseradish peroxidase (HRP)-labeled goat anti-rabbit IgG. Color development was performed using a DAB color development kit (ZhongShan Biotech). Positively stained areas were quantified using ImageJ 8.0 (Image software, Bethesda, MD, United States).

### 2.15 Safranin O Fast Green staining

The sections were deparaffinized using xylene and rehydrated in a graded alcohol series. After washing with water, the slices were submerged in Fast Green solution for 3–5 min. They were then rinsed with a 1% HCl-alcohol solution, rewashed, and then immersed in Safranin O solution for 30 s. Finally, the sections were quickly dehydrated in two changes of pure ethanol. The mounting process involved sequentially immersing the slices in n-butanol and xylene to achieve transparency. The slices were sealed using neutral gum. Images were captured under a light microscope (Olympus, Tokyo, Japan).

### 2.16 Tartrate-resistant acid phosphatase (TRAP) staining

Osteoclastogenesis in mouse jawbone sections was observed using a TRAP staining kit in accordance with the kit’s specifications (Sigma-Aldrich, St Louis, MO, United States). Positive staining was captured under a light microscope (Olympus, Tokyo, Japan).

### 2.17 Statistical analysis

Data were expressed as mean ± standard deviation, and statistical analyses were performed using the SPSS 23.0 software. One-way ANOVA was employed to compare data following a normal distribution. Non-normally distributed data were compared using the Kruskal–Wallis test. P < 0.05 was considered statistically significant.

## 3 Results

### 3.1 Upregulation of lipid metabolism-related genes and pathways in periodontitis

**FIGURE 2:**
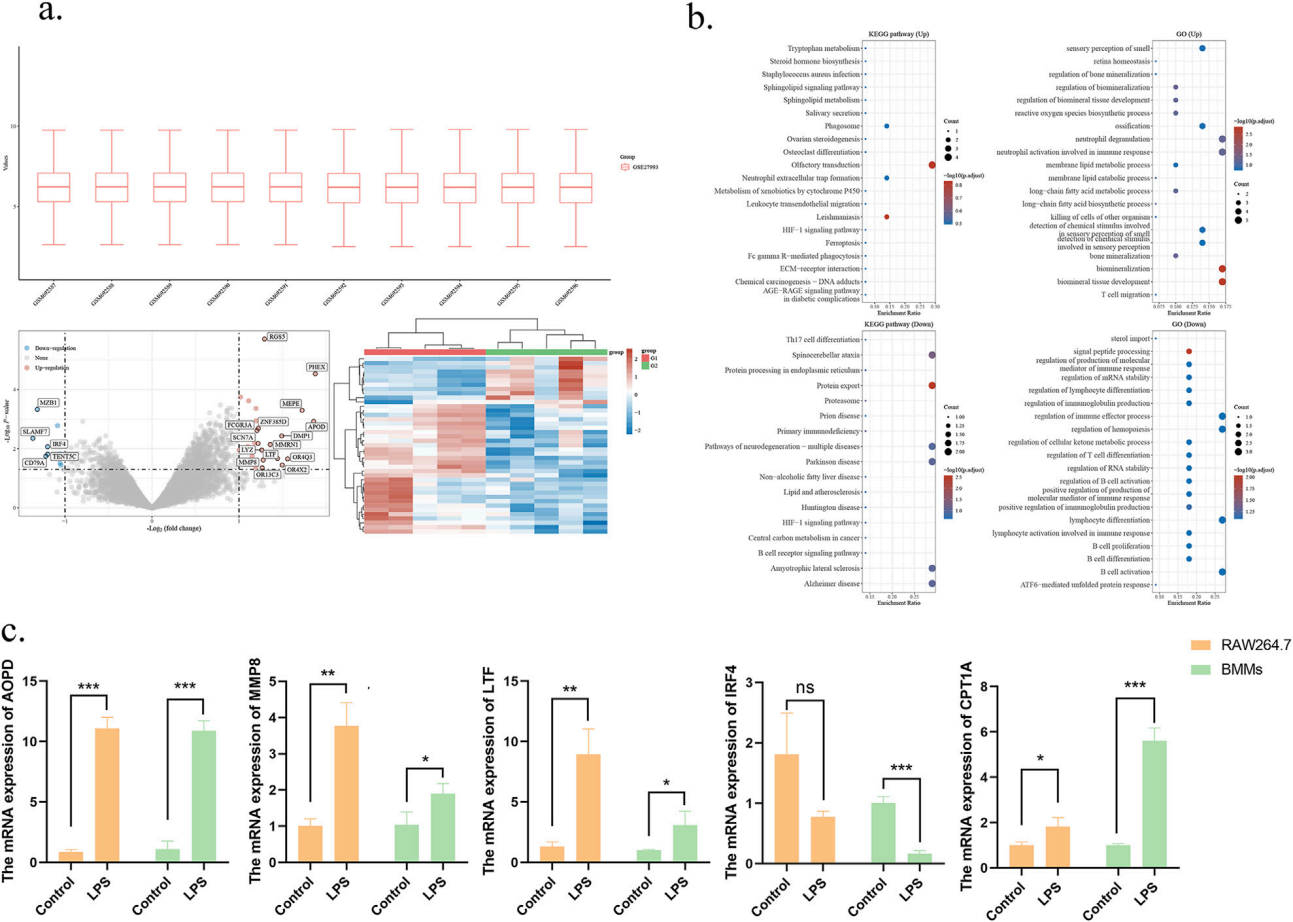
Lipid metabolism-related expressed genes in periodontitis were analyzed using bioinformatics approaches. **(a)** Cluster analysis of differentially expressed mRNAs between the normal and periodontitis groups, and volcano plots were used to analyze the differential expression of mRNAs between groups. Red indicates upregulation and blue color indicates downregulation. **(b)** Target genes were subjected to Gene Ontology (GO) annotation and Kyoto Encyclopedia of Genes and Genomes (KEGG) pathway analysis. **(c)** The expression of genes related to lipid metabolism between the normal and inflammatory groups was verified by qRT-PCR. *p < 0.05; **p < 0.01; ***p < 0.001.

The gene expression matrix (GSE27993) was downloaded, and differential analysis was performed to identify differentially expressed genes (DEGs) before and after the induction of periodontitis. To examine the biological roles of these DEGs, functional enrichment analyses were carried out on the upregulated and downregulated genes using R software. These analyses included Gene Ontology (GO) categories for Biological Process (BP), Molecular Function (MF), and Cellular Component (CC), as well as Kyoto Encyclopedia of Genes and Genomes (KEGG) pathway analyses. The results of GO enrichment analysis revealed that biological processes were predominantly enriched in the membrane lipid metabolic process, long-chain fatty acid metabolic process, and other related processes. Similarly, lipid metabolism-related signaling pathways, such as steroid hormone biosynthesis, the sphingolipid signaling pathway, and sphingolipid metabolism, were significantly enriched. Meanwhile, the genes APOD, MMP8, and LTF, which are involved in lipid metabolism, exhibited increased expression levels, whereas the expression level of the IRF4 gene was lower compared to the normal control group (Figures 2a,b). Furthermore, the differential expression levels of four lipid metabolism-related genes were further validated in an *in vitro* periodontitis-mimicking environment using RAW264.7 and BMMs cells stimulated with *P. gingivalis*-LPS. As anticipated, the results of qRT-PCR were consistent with those in the GSE27993 dataset. The results indicated that the expression of three genes, namely, APOD, MMP8, and LTF, was upregulated, whereas that of IRF4 was downregulated in the non-inflammatory state compared to the normal group, consistent with the findings of the bioinformatics analysis (Figure 2c).

Considering that the aforementioned analysis enriched multiple lipid metabolic pathways, it can be inferred that APOD, MMP8, and LTF regulate inflammation and oxidative stress by participating in FAO processes and contribute to chronic inflammatory diseases such as obesity (Fyfe-Desmarais et al., 2023; Yu and Li, 2022). However, no studies have investigated the relationship between FAO and inflammation in periodontitis. At the same time, as a key regulator of M2 macrophages, the downregulation of IRF4 expression may impair the function of M2 macrophages, given that the anti-inflammatory function of M2 macrophages is dependent on fatty acid oxidation (FAO). Since *CPT1A* is a crucial enzyme in FAO, the results suggest that the expression of *CPT1A* may be altered in periodontitis. Based on these findings, *CPT1A* was selected for further analysis to explore its potential role in periodontitis.

### 3.2 Effects of ETO and P.gingivalis-LPS on the inflammatory factor levels, and activation of signaling pathways in RAW264.7 cells

**FIGURE 3:**
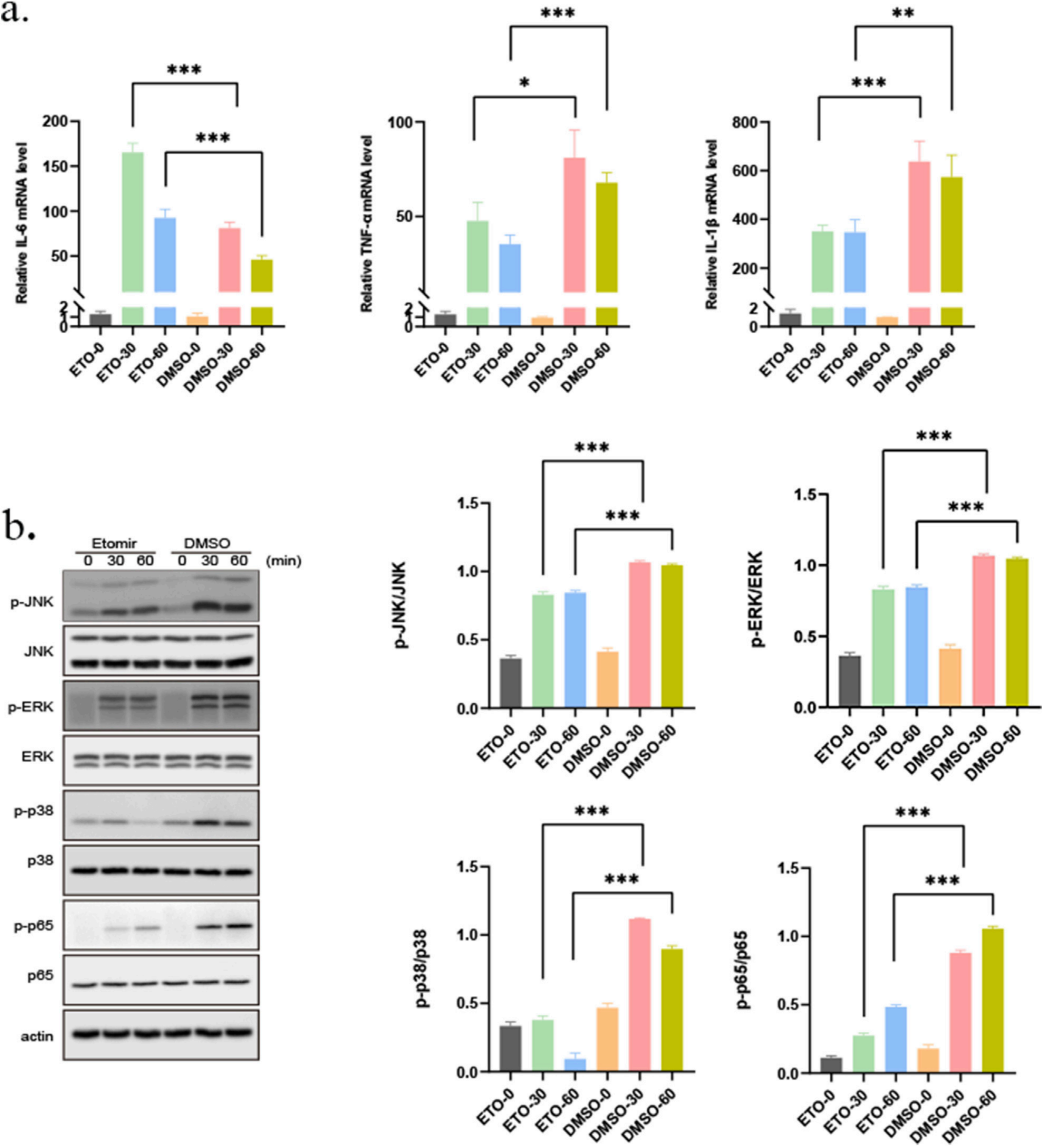
Effect of ETOMOXIR on the gene expression and activation of pathways in an inflammatory environment. **(a)** TNF-α, IL-6 and IL-1β mRNA expression levels in RAW264.7 cells across groups (0: without treatment, ETO-30: 30 min 1 μg/mL ETO+1 μg/mL P. gingivalis-LPS; ETO-60: 60 min 1 μg/mL ETO+ 1 μg/mL P. gingivalis-LPS; DMSO-30: 30 min DMSO+ 1 μg/mL P. gingivalis-LPS; DMSO-60: 60 min DMSO+1 μg/mL P. gingivalis-LPS). **(b)** Western blot analysis of the MAPK/P65 signaling pathway across treatment groups (0: without treatment, ETO-30: 30 min 1 μg/mL ETO+ 1 μg/mL P. gingivalis-LPS; ETO-60: 60 min 1 μg/mL ETO+1 μg/mL P. gingivalis-LPS; DMSO-30: 30 min DMSO+ 1 μg/mL P. gingivalis-LPS; DMSO-60: 60 min DMSO+ 1 μg/mL P. gingivalis-LPS). *p < 0.05; **p < 0.01; ***p < 0.001.

To ensure that the effects observed were attributable to the pharmacological action of ETO rather than cytotoxicity, a CCK-8 assay was performed. As illustrated in Supplementary Figure S1, the results confirmed that ETO concentrations below 1 μg/mL did not affect macrophage proliferation and viability. Then, RAW264.7 cells were pre-incubated with 1 μg/mL ETO for 30 min or 60 min. After changing the medium, live P. gingivalis-LPS was added, and the cells were co-cultured for 3 h. Interestingly, stimulation with P. gingivalis-LPS significantly increased the levels of inflammatory markers in macrophages, with IL-6 mRNA levels increasing by 50 folds (p < 0.0001), TNF-α mRNA levels increasing by 20 folds (p < 0.0001), and IL-1β mRNA levels increasing by 20 folds (p < 0.0001), indicating a pronounced trend toward inflammatory polarization, as displayed in Figures 3a–c. At the same time, the expression of *CPT1A* was significantly inhibited in ETO pre-incubated group compared to the DMSO group (Supplementary Figure S2).

Besides, the results of Western blot analysis unveiled that ETO inhibited the activation of the Erk, JNK, and p38 signaling pathways (Figure 3b). In addition, a similar trend was noted in NF-κB. Specifically, NF-κB phosphorylation levels were significantly increased following stimulation with *P. gingivalis*-LPS. Conversely, ETO strongly attenuated NF-κB activation, resulting in reduced inflammatory responses in macrophages. The reduced expression levels of inflammatory cytokines in P. gingivalis-LPS-induced RAW264.7 cells suggested that ETO may inhibit the release of inflammatory cytokines by immune cells in periodontitis.

### 3.3 *SIRT2* deacetylase binds to the *CPT1A* promoter to regulate its expression

**FIGURE 4:**
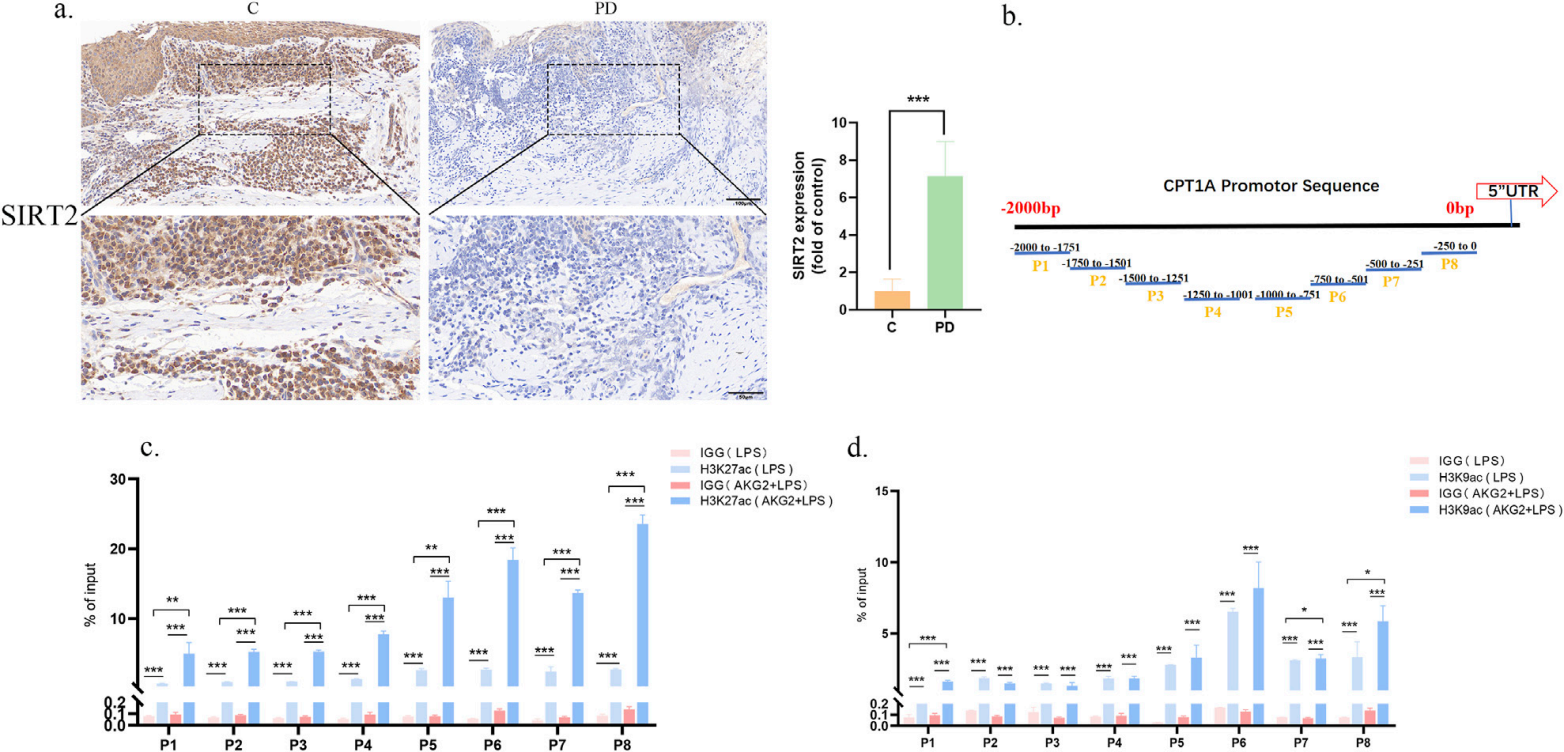
*SIRT2* regulates *CPT1A* expression through deacetylation. **(a)** Immunohistochemical staining of *SIRT2* in patients C: control, PD: periodontitis. **(b)** The *CPT1A* promoter region was divided into 8 segments to design primers. **(b)** H3K27ac enrichment in the *CPT1A* promoter region was assessed in the LPS and the LPS + AGK2 groups and compared with the IgG pull-down of total protein. **(c,d)** H3K9ac enrichment in the *CPT1A* promoter region was evaluated in the LPS and the LPS + AGK2 groups and compared with the IgG pull-down of total protein. *p < 0.05; **p < 0.01; ***p < 0.001.

Epigenetic modifications can mediate the interaction between chromatin structure and gene expression. Histone acetylation plays a vital role in the development of periodontitis (Li et al., 2023; Bae, 2017). Previous studies described that P. gingivalis-LPS elevated the acetylation level of H3K27 (Shi et al., 2024). In primary hepatocytes, ethanol-induced hepatic histone H3K9 deacetylation at the CPT-1A promoter downregulates CPT-1A gene expression (Donde et al., 2020). Prior investigations have reported that an increase in *SIRT2* levels in mice was associated with an change in *CPT1A* expression (Helsley et al., 2023). Immunohistochemical analysis revealed that the expression of *SIRT2* in gingival tissues from periodontitis patients was significantly lower than in normal gingival tissues. This aligns with the expression trend of *SIRT2* at the cellular level. *SIRT2* expression decreased following stimulation with P. gingivalis LPS, whereas it increased after pretreatment with ETO (Supplementary Figure S3). Therefore, we hypothesize that *SIRT2* may play a crucial role in the onset and progression of periodontitis by deacetylating *CPT1A* (Figure 4a). To examine the effects of post-translational histone modifications at the *CPT1A* promoter in response to *P. gingivalis*-LPS, a Chromatin immunoprecipitation (ChIP) assay was performed. Initially, ChIP analysis was conducted on the promoter regions of the CPT-1A gene (Figure 4b). In addition, the status of histone H3 lysine 9 acetylation (H3K9Ac) and H3 lysine 27 acetylation (H3K27Ac), which play a key role in the transcriptional activation of gene expression, was examined. Then, RAW264.7 cells were treated with AGK2, a histone deacetylase inhibitor that specifically inhibits *SIRT2* expression. The results of ChIP-qPCR demonstrated that H3K27ac enrichment levels were elevated to varying degrees across all regions, while H3K9ac enrichment levels were observed in the P1, P6, and P8 regions (Figures 4c,d). AGK2 pretreatment not only increased *CPT1A* levels but also significantly increased H3K27Ac levels by approximately 2.5-fold in regions P6, P7, and P8. These results collectively suggest that *SIRT2* binds to and deacetylates *CPT1A* that can decreased the inflammation. Furthermore, the *SIRT2* gene can be positively regulated to restore the expression of *CPT1A*, thereby serving as a transcriptional co-repressor.

### 3.4 ETO treatment ameliorates experimental periodontitis in mice

**FIGURE 5:**
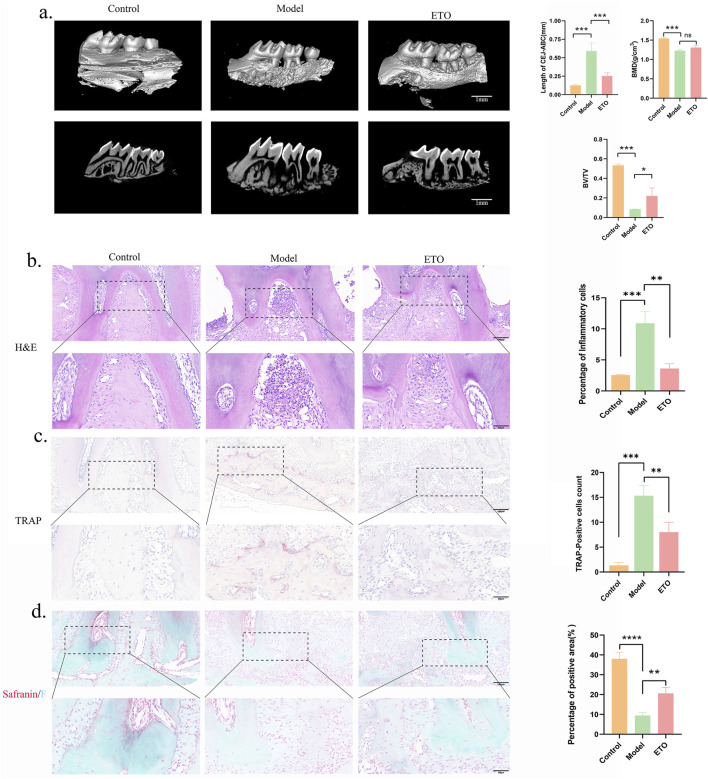
Etomoxir treatment mitigates periodontitis-induced alveolar bone resorption. **(a)** Micro-CT (Scale bar = 1 mm); Analysis of BMD, BV/TV, and the distance between CEJ and ABC. **(b-d)** H&E staining **(b)**, TRAP staining **(c)** and Safranin O Fast Green staining **(d)** results of the maxillary region. (Scale bar = 100 μm) *p < 0.05; **p < 0.01; ***p < 0.001.

To validate our findings *in vivo*, a ligature-induced experimental periodontitis model was constructed in mice. C57BL/6 mice with experimental periodontitis were gavaged with or without ETO daily for 7 days. The distance between the CEJ and ABC, which reflects the degree of alveolar bone loss, was measured to quantify the degree of periodontal bone resorption. Moreover, micro-CT scanning was performed to quantify and analyze alveolar bone loss. The results indicated that the degree of bone resorption was higher in the Model group compared to the Control group, indication the successful construction of the experimental periodontitis model. However, the progression of alveolar bone resorption was attenuated by ETO. More importantly, changes in BV/TV and BMD across treatment groups were consistent with the observed bone resorption (Figures 5a,b). H&E staining delineated that alveolar bone destruction was more severe in the P group compared to the ETO group. Meanwhile, inflammatory infiltration was more pronounced in the Model group compared to the ETO group. Furthermore, the number of TRAP-positive osteoclasts was lower in the ETO group. Safranin O Fast Green staining displayed less intense solid green staining, greater loss of bone tissue, and a narrower range and lighter-stained collagen fiber in the Model group compared to the ETO group, as depicted in Figure 5c. These findings collectively indicate that FAO plays a crucial role in the development of experimental periodontitis and that ETO can effectively prevent experimental periodontitis by inhibiting *CPT1A* expression. Taken together, these results signaled that ETO may mitigate periodontal inflammation by limiting alveolar bone resorption.

### 3.5 ETO treatment reduces experimental periodontal tissue inflammation

**FIGURE 6:**
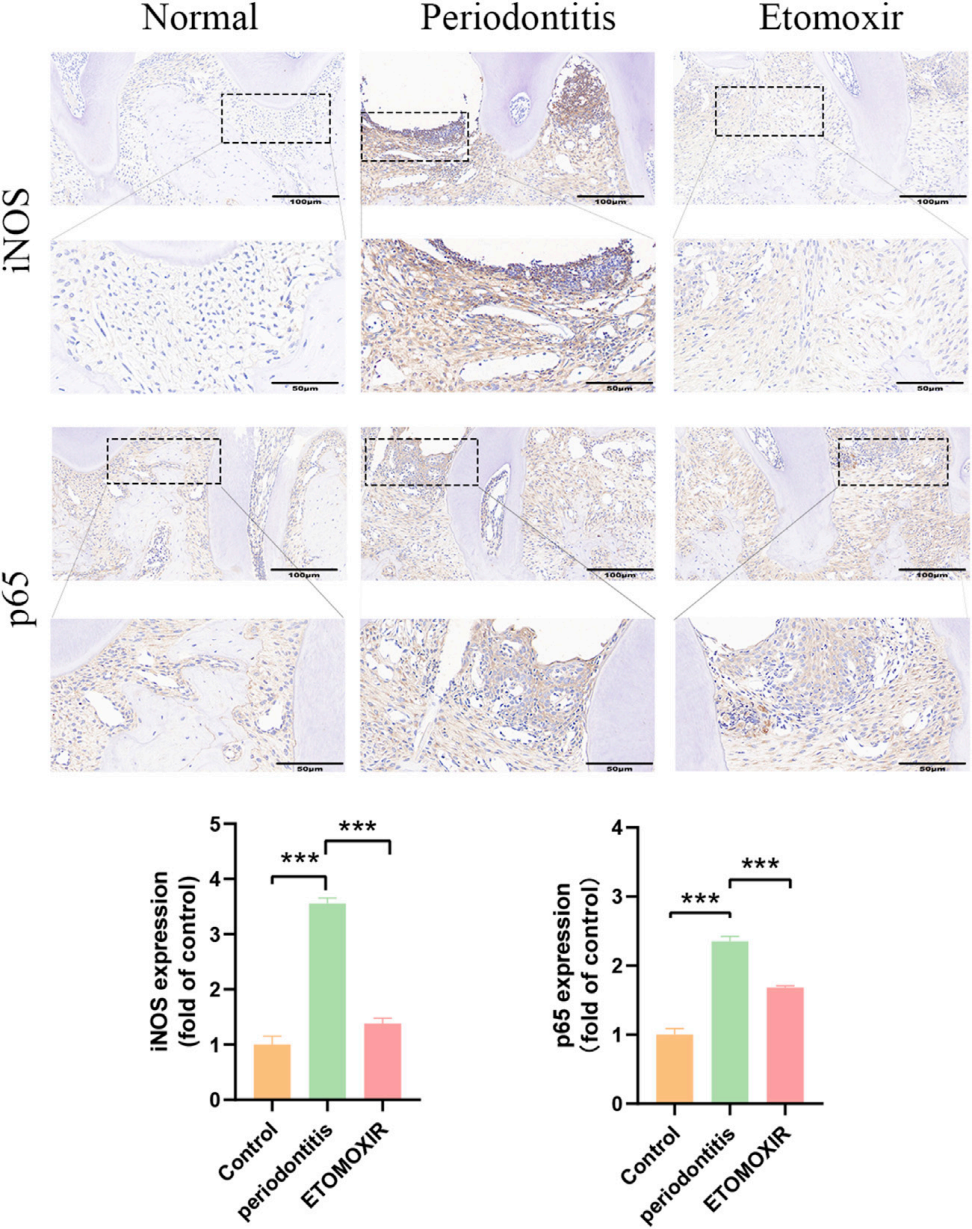
Immunohistochemical staining of iNOS, p65 in the periodontal tissue of mouse maxillae (Scale bar = 100 µm and Scale bar = 50 µm). *p < 0.05; **p < 0.01; ***p < 0.001.

Further experiments were conducted to explore the mechanistic connection between ETO and periodontitis. Specifically, the effects of ETO on inflammation in gingival tissues during experimental periodontitis were investigated. iNOS is strongly linked to the activation of M1-type macrophages, an essential feature of inflammatory macrophages. Additionally, the progression of periodontal inflammation is driven by the activation of NF-κB signaling molecules. We postulate that ETO may potentially delay the progression of experimental periodontitis by inhibiting the activation of inflammatory signals. Immunohistochemical staining of periodontal tissues uncovered that the ETO group showed a significant decrease in *CPT1A*, demonstrating the effectiveness of the inhibitor (Supplementary Figure S4). And the expression levels of iNOS and p65 were significantly higher in the periodontal tissues of mice in both the Model group and ETO group compared to the Control group. Furthermore, after treatment, the levels of iNOS and p65 were lower in the ETO group compared to the Model group (Figure 6). Meanwhile, the expression of the M2 macrophage marker CD163 was significantly increased after ETOMOXIR treatment (Supplementary Figure S5). These results conjointly suggest that ETO can delay the progression of experimental periodontitis by down-regulating the expression of p65 and the pro-inflammatory mediator iNOS.

## 4 Discussion

Periodontitis is a prevalent oral disease that significantly impacts health and overall wellbeing. Nevertheless, our understanding of the pathogenesis of periodontitis remains limited. Macrophages, key components of the immune system, have been extensively studied and have been established to undergo pro-inflammatory M1 polarization following exposure to LPS derived from *Porphyromonas gingivalis*. As a result, RAW264.7 cells have been utilized *in vitro* to investigate the molecular mechanisms underlying periodontitis (Zhang B. et al., 2021; Yang, et al., 2021; Kong et al., 2019). Recent studies pointed out that high-fat diets may be correlated with periodontitis (Schenkein et al., 2020). Indeed, high-fat diets rich in saturated fatty acids and cholesterol can activate pro-inflammatory factors (TNF-α, IL-6, IL-8, IL-1β) through multiple pathways (Phu et al., 2023). In addition, a high-fat state may change the composition of oral microorganisms by altering the types and numbers of bacteria in the oral cavity, thereby increasing the risk of periodontitis (Cani et al., 2007; Qin et al., 2018). This study utilized bioinformatics analysis and qRT-PCR to identify differentially expressed genes between patients with periodontitis and healthy patients. The findings exposed that the primary pathways of interest were associated with long-chain fatty acid metabolism. Furthermore, key molecules that exhibited differential expression, namely, IRF4, APOD, PHEX, and MEPE, participated in lipid metabolism and osteogenesis. Thus, we propose a significant association between lipid metabolism and periodontitis.

Lipid metabolism is heavily reliant on FAO, a crucial metabolic pathway. Previous studies have determined that the APOD gene, which is highly expressed in periodontitis, inhibits FAO and concomitantly mitigates oxidative stress (Han et al., 2023). The IRF4 gene, which is significantly downregulated in periodontitis, confers resistance to obesity and enhances insulin sensitivity following specific knockout in mice. Therefore, we speculate that excessive FAO occurs in periodontitis and triggers compensatory changes in the expression levels of relevant genes. Suppressing the activity of *CPT1A* during FAO has been found to inhibit the development of liver fibrosis and nonalcoholic fatty liver disease (Weber et al., 2020). In addition, *CPT1A* appears to be a candidate therapeutic target in various cancers, including lung cancer, breast cancer, etc., (Ma et al., 2024; Altea-Manzano et al., 2023; Tang et al., 2022). Nonetheless, the role of *CPT1A* has not been specifically explored in periodontitis. To address this gap, P. gingivalis-LPS was employed to induce macrophage activation and establish an experimental inflammation model in the laboratory. This model was subsequently utilized to investigate the role of *CPT1A*, a crucial enzyme involved in FAO, and its association with different inflammatory processes. The results demonstrated that the NF-KB and MAPK pathways were activated in cells exposed to P. gingivalis-LPS compared to the normal group. Additionally, the expression levels of the inflammatory cytokines TNF-α, IL-6, and IL-1β were increased, an effect that was reversed following the introduction of a *CPT1A* inhibitor. Earlier studies have shown that increased levels of proinflammatory cytokines in the alveolar bone stimulate the formation of osteoclasts and facilitate bone resorption. Additionally, previous studies have documented that the activation of the MAPK signaling pathway in various immune cells modulates the progression of periodontal disease and the loss of alveolar bone (Kumar et al., 2021; Wang et al., 2020), suggesting that *CPT1A* is involved in the inflammatory response of macrophages *in vitro*.

Sirtuins are nicotinamide adenine dinucleotide (NAD+)-dependent histone deacetylases that mediate energy metabolism and aging and directly or indirectly regulate lipid metabolism. Previous studies have demonstrated that Sirt1, Sirt3, and Sirt5 can induce FAO by deacetylating and activating key enzymes (Zhang et al., 2022; Naia et al., 2021; Qu et al., 2024). Nonetheless, the correlation between *SIRT2* and FAO remains unknown, particularly in the context of periodontitis. Previous studies have outlined that a reduction in *SIRT2* levels may be linked to aging, whilst its deacetylation is positively associated with longevity and delayed aging. These factors play a significant role in the progression of periodontal diseases. Conversely, the promoter region of *CPT1A* can undergo various histone post-translational changes, including phosphorylation, acetylation, ubiquitination, etc., which in turn impact fatty acid oxidation. In a mouse model of obesity induced by a high-fat diet, the level of H3K9me was significantly increased in the promoter region of *CPT1A* (Su et al., 2023), which subsequently downregulated *CPT1A* expression and thus hindered fatty acid oxidation. Histone deacetylase (HADS) inhibitors enhance the transcription of the *CPT1A* gene by promoting its expression through acetylation (Donde et al., 2020; Kirpich et al., 2012), thereby increasing the oxidative metabolism of fatty acids, potentially improving the energy function of liver cells. According to a previous study, individuals with periodontitis have lower levels of several HDACs and a significantly higher level of H3K9ac in afflicted tissues and saliva (Suzuki and Yamada, 2022). These fluctuations exacerbate inflammation, impair new bone formation, and induce pathological deterioration. Therefore, we hypothesize that *CPT1A* histone acetylation may play a central role in the development of periodontitis. Specifically, H3 acetylation at specific lysine residues (H3K14, H3K9, and H3K27) is associated with transcriptional activation, while deacetylation is linked to transcriptional repression. Histone acetylation modifications are typically associated with gene activation, whereas deacetylation modifications reduce the level of acetylation at the site, thereby inhibiting gene expression. Previous studies have evinced that *SIRT2* is significantly correlated with *CPT1A* expression in metabolic diseases and that the *in vitro* overexpression of ketohexokinase-C (KHK-C) decreases the level of *SIRT2* while promoting *CPT1A* acetylation (Helsley et al., 2023). To investigate the relationship between *CPT1A* histone acetylation and periodontitis, the present study explored the correlation between *SIRT2* and *CPT1A*, as well as the presence of acetylation modifications in the promoter region of *CPT1A* in an *in vitro* model using Chip-qPCR. The results demonstrated that the acetylation of H3K9 and H3K27 at the *CPT1A* promoter could be influenced by the depletion of *SIRT2* by *SIRT2*-specific inhibitors. The activity of *CPT1A* was enhanced by elevated promoter expression, which promoted fatty acid oxidation, in line with the findings observed in earlier experiments.

To further confirm the presence of overactive FAO in periodontitis, the functional role of *CPT1A* was examined. Herein, ETO, a *CPT1A*-specific inhibitor, effectively mitigated inflammatory bone loss. Importantly, a mouse model of periodontitis was constructed using conventional silk ligation, wherein plaque formed around the ligature wire at the ligation site, thereby promoting inflammation and the destruction of periodontal tissues within a few days (Shen et al., 2020; Kawahara et al., 2009; Zhang Z. et al., 2021). Given that the pathologic manifestations in patients with periodontitis are phenotypically similar to those in the constructed mouse model, this approach has been widely used to examine the mechanisms underlying periodontitis and explore novel targets for its treatment. Notably, ETO played a dual role in alleviating ligation-induced periodontitis. Firstly, it attenuated alveolar bone resorption in a model of ligation-induced periodontitis. In line with previous studies that reported increased osteoclastogenesis and collagen resorption as hallmarks of bone destruction in a ligation-induced periodontitis model, the number of osteoclasts around the alveolar bone was significantly increased in the periodontitis model of this study (Taira et al., 2019). Of note, the number of osteoclasts and the degree of collagen fiber loss in the corresponding areas was significantly lower in the ETO-treated group compared to the periodontitis group. Noteworthily, micro-CT delineated that *CPT1A* inhibition decreased the number of osteoclasts around the maxilla and prevented bone loss. On the other hand, the results of immunohistochemical staining showed that ETO inhibited NF-κB and iNOS activation in oral periodontal tissues, accompanied by decreased periodontal inflammation and inflammatory cell infiltration.

Based on these findings, we theorize that *CPT1A*, a key mediator in FAO, is implicated in the progression of periodontitis. In addition, *SIRT2* can downregulate *CPT1A* expression by deacetylating its *CPT1A* promoter, thereby partially delaying the progression of periodontitis. However, it is crucial to recognize that this study exclusively focused on the management of periodontal disease progression. Indeed, some limitations of this study merit acknowledgment, given the differences between human periodontitis and the mouse model of inflammation. Moreover, periodontal disease models cannot comprehensively replicate the complexity present *in vitro* conditions. Thus, the role of *CPT1A* as a potential target for bone regeneration in periodontitis-induced inflammatory bone loss remains uncertain. Further research is warranted to elucidate the therapeutic role of *SIRT2* in attenuating periodontal inflammation and explore safe and effective delivery methods for these targeted drugs.

## 5 Conclusion

**FIGURE 7:**
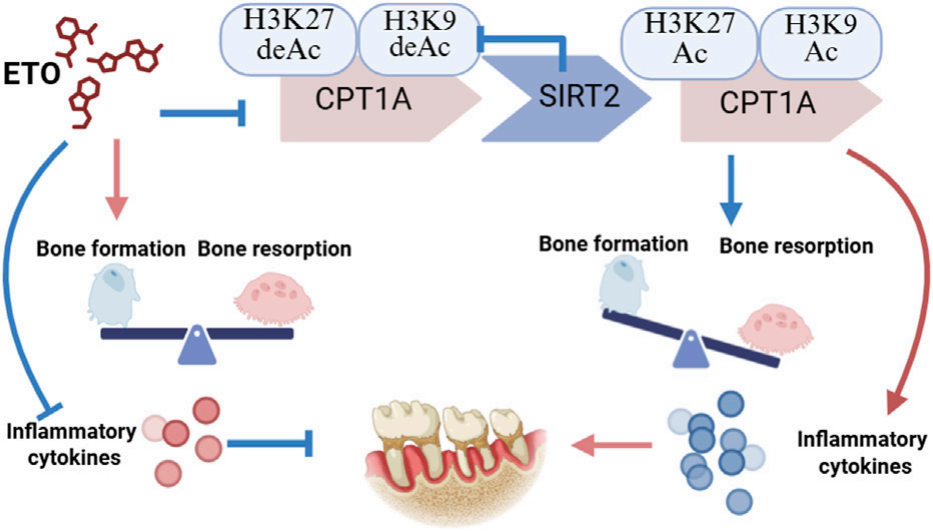
*SIRT2* binds to and deacetylates *CPT1A*. This process inhibits the differentiation of osteoclasts and alleviates inflammation in periodontal tissues during periodontitis progression.

To the best of our knowledge, this is the first study to demonstrate that the deacetylation of *CPT1A* histones by *SIRT2* may regulate the progression of periodontitis by suppressing the activation of the MAPK and NF-κB signaling pathways, thereby inhibiting osteoclast differentiation and minimizing the production of inflammatory factors (Figure 7).

## Data Availability

The original contributions presented in the study are included in the article/Supplementary Material, further inquiries can be directed to the corresponding authors.
