# Supplementary material for: SIRT2-dependent CPT1A deacetylation in macrophages inhibits periodontitis
Source: Front Pharmacol. 2025 Jun 18;16:1574141. doi: 10.3389/fphar.2025.1574141 (PMC12213876; doi:10.3389/fphar.2025.1574141)
Supplement: Supplementary file 1 [file DataSheet1.docx]

**
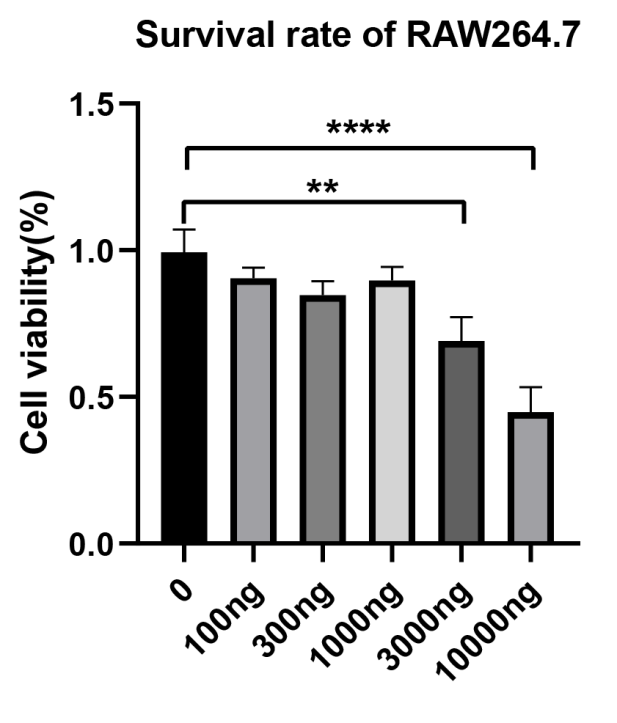
**

**Supplementary Figure 1.** The effect of ETOMOXIR on RAW264.7 cells. Cytotoxicity of ETOMOXIR at concentrations of 100–10000 μg/mL.


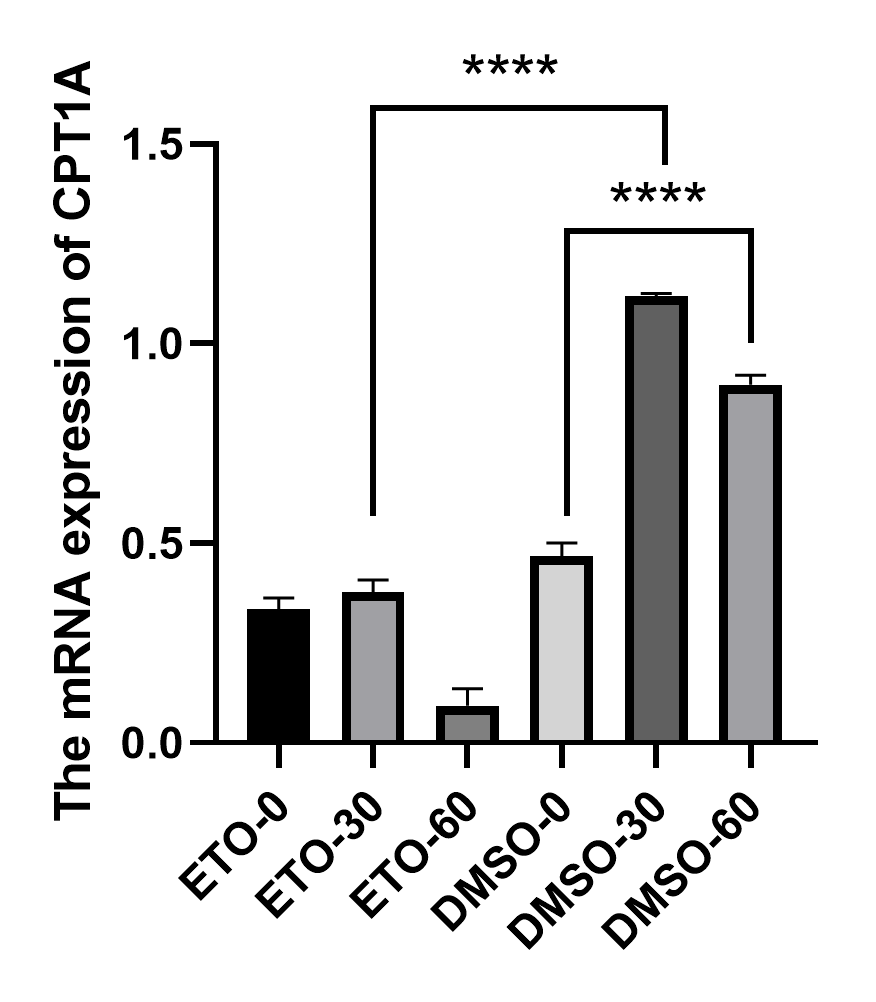


**Supplementary Figure 2.** CPT1A mRNA expression levels in RAW264.7 cells across groups (0: without treatment, ETO-30: 30 min 1 µg/mL ETO+1 µg/mL *P.gingivalis*-LPS; ETO-60: 60 min 1 µg/mL ETO+ 1 µg/mL *P.gingivalis*-LPS; DMSO-30: 30 min DMSO+ 1 µg/mL *P.gingivalis*-LPS; DMSO-60: 60 min DMSO+1 µg/mL P.gingivalis-LPS)


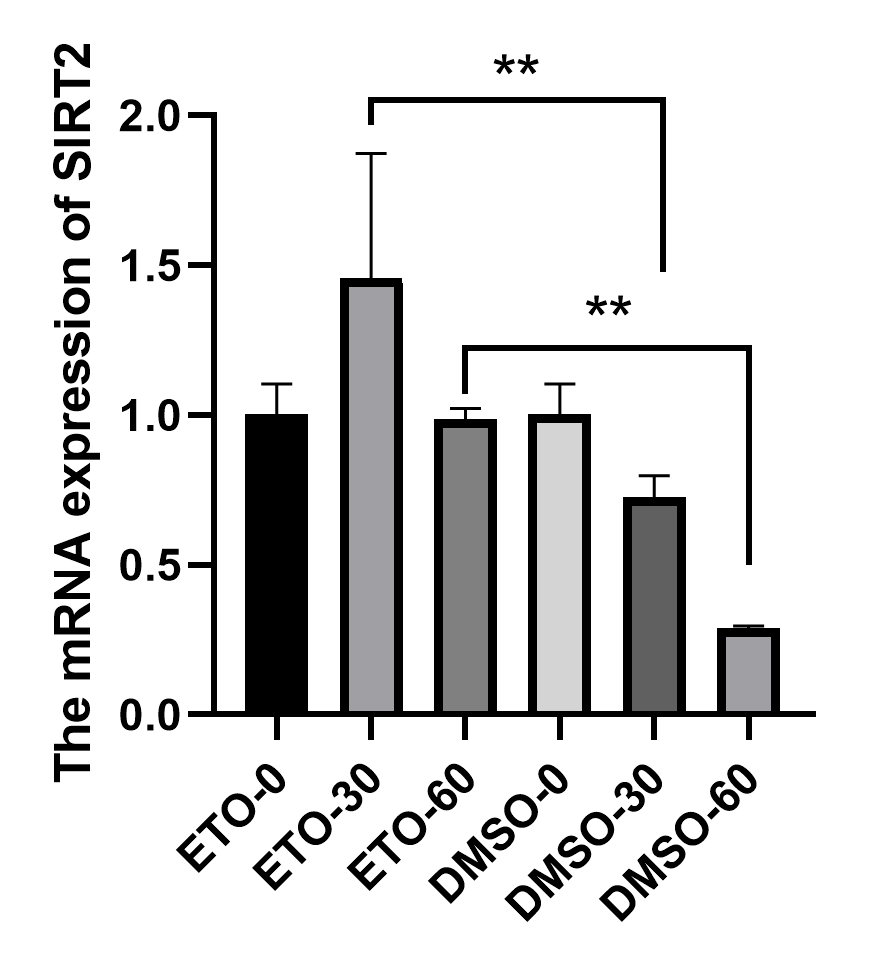


**Supplementary Figure 3.** SIRT2 mRNA expression levels in RAW264.7 cells across groups (0: without treatment, ETO-30: 30 min 1 µg/mL ETO+1 µg/mL *P.gingivalis*-LPS; ETO-60: 60 min 1 µg/mL ETO+ 1 µg/mL *P.gingivalis*-LPS; DMSO-30: 30 min DMSO+ 1 µg/mL *P.gingivalis*-LPS; DMSO-60: 60 min DMSO+1 µg/mL P.gingivalis-LPS)


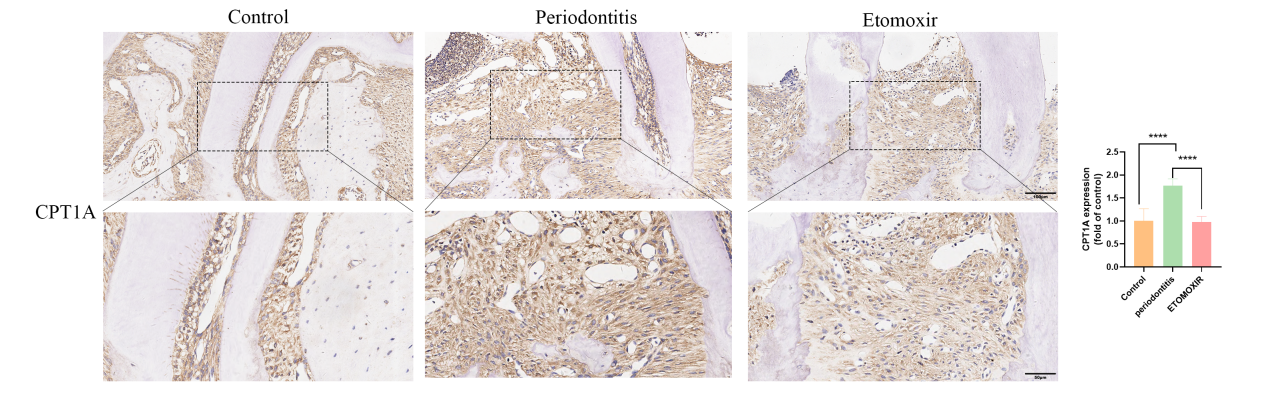


**Supplementary Figure 4.** Immunohistochemical staining of CPT1A in the periodontal tissue of mouse maxillae (Scale bar = 100µm and Scale bar = 50 µm). * p < 0.05; ** p < 0.01; *** p < 0.001.**** p < 0.001.


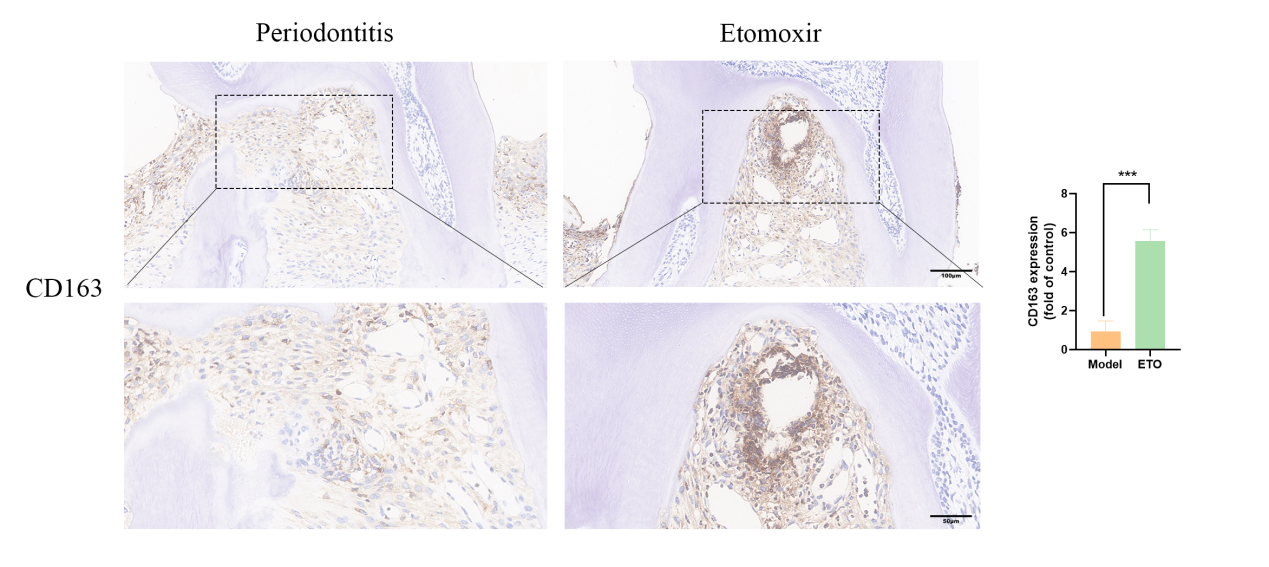


**Supplementary Figure 5.** Immunohistochemical staining of CD163 in the periodontal tissue of mouse maxillae (Scale bar = 100µm and Scale bar = 50 µm). * p < 0.05; ** p < 0.01; *** p < 0.001.**** p < 0.001.
